# Nonenzymatic function of Aldolase A downregulates miR-145 to promote the Oct4/DUSP4/TRAF4 axis and the acquisition of lung cancer stemness

**DOI:** 10.1038/s41419-020-2387-2

**Published:** 2020-03-18

**Authors:** Yu-Chan Chang, Yi-Fang Yang, Jean Chiou, Hsing-Fang Tsai, Chih-Yeu Fang, Chih-Jen Yang, Chi-Long Chen, Michael Hsiao

**Affiliations:** 1 Genomics Research Center, Academia Sinica, Taipei, 115 Taiwan; 2 Department of Medical Education and Research, Kaohsiung Veterans General Hospital, Kaohsiung, Taiwan; 3 Department of Internal Medicine, Kaohsiung Municipal Ta-Tung Hospital, Kaohsiung Medical University, Kaohsiung, Taiwan; 4 Department of Pathology, Taipei Medical University Hospital, Taipei Medical University, Taipei, 110 Taiwan; 5 Department of Pathology, College of Medicine, Taipei Medical University, Taipei, 110 Taiwan; 6 Department of Biochemistry, College of Medicine, Kaohsiung Medical University, Kaohsiung, 807 Taiwan

**Keywords:** Cancer metabolism, Cancer stem cells

## Abstract

Drug resistance remains a serious issue of clinical importance and is a consequence of cancer stemness. In this study, we showed that the level of Aldolase A (ALDOA) expression is significantly associated with the IC50 value of chemotherapy drugs in lung cancer. Our data revealed that ALDOA overexpression resulted in a significant increase of lung tumor spheres. The use of ingenuity pathway analysis (IPA) resulted in the identification of *POU5F1* (Oct4) as the leading transcription factor of ALDOA. We observed high expression of ALDOA, Oct4 and stemness markers in collected spheroid cells. DUSP4 and TRAF4 were confirmed as major downstream targets of the ALDOA-Oct4 axis. Knockdown of these molecules significantly decreased the stemness ability of cells. In addition, we investigated whether miR-145 targets the 3′-UTR of Oct4 and is regulated by ALDOA due to the involvement of ALDOA in glycolysis and metabolic reprogramming. Furthermore, we constructed several mutant forms of ALDOA that disrupted its enzymatic activity and showed that they still induced significant in vitro sphere formation and in vivo tumorigenicity. These results demonstrated that ALDOA-mediated spheroid formation is independent of its enzymatic activity. In the clinical component, we also showed that the combination of ALDOA and TRAF4 or DUSP4 is positively correlated with poor overall survival in a xenograft model and cancer patients through immunohistochemical analyses. The results of our study revealed novel functional roles of ALDOA in inducing cancer stemness via the inhibition of miR-145 expression and the activation of Oct4 transcription. These findings offer new therapeutic strategies for modulation of lung cancer stemness to enhance chemotherapeutic responses in lung cancer patients.

## Introduction

The recurrence and metastasis of malignant tumors lead to poor outcomes in patients and are important issues in the clinical setting^1^. Cancer stem cells are an important issue in cancer treatment. Cancer stem cells are viewed as a side population of cancer cells that have a self-renewal function and pluripotency ability compared with those of typical cancer cells^2^. Importantly, previous reports have showed that cancer stem cells form spheroids that combat the efficacy of chemotherapy and radiotherapy^3,4^. Recently, scientists have investigated several key factors for stemness and spheroid formation, including *POU5F1* (Oct4), *KLF4*, *SOX2* and *Nanog*, the activation of which induces several canonical pathways involved in tumor relapse after treatment^5–7^. Understanding of the unique characteristics and vulnerabilities is expected to enable the development of new strategies for targeting cancer stem cells to prolong patient survival and overcome cancer metastasis.

To better understand the role of cancer stem cells in cancer recurrence, recent studies have focused on blocking signaling pathways or investigating genetic alterations of cancer stem cells^8^. For example, epithelial-mesenchymal transition (EMT) is important in the initiation of metastasis of cancer stem cells^9^. In addition, the Wnt^10,11^ and Notch pathways^12,13^ are crucial in activating the proliferation and stemness abilities of cells. Cancer stem cells not only depend on their specific abilities but also on several events to support and maintain the environment and conditions for these cells. Moreover, the metabolic events of cancer stem cells demonstrate their unique roles, depending on the various cancer types^14^. For example, lung sphere-forming cells and glioma spheres have an oxidative phosphorylation (OXPHOS) phenotype^15,16^. In contrast, liver and breast cancer stem-like cells (CD133^+^/CD49f^+^ and CD44^+^CD24^low^EPCAM^+^, respectively) use a glycolytic pathway rather than OXPHOS^17,18^. Therefore, we propose that glycolytic enzymes play a key role in regulating cancer stemness-related factors or metabolite production to promote cancer stemness.

In this study, we screened all of the glycolytic enzymes in the assayed lung cancer cell lines at the mRNA level. In addition, we obtained the corresponding IC50 values of chemotherapy drugs from an online website. The results showed that ALDOA was significantly and positively correlated with the IC50 of cisplatin in lung cancer cells. To investigate the characteristics of ALDOA in lung cancer stem cells, we established two-way models, several of which indicated that ALDOA indeed promotes the sphere-forming ability of lung cancer cells in vitro and in vivo in the absence of aldolase enzyme activity. Via transcriptomic analysis, we observed that ALDOA suppresses miR-145 expression, which activates pluripotency factors and the downstream DUSP4/TRAF4 axis. This signature was also shown to correlate with patient survival in lung cancer clinical cohorts, as demonstrated by an in silico statistical analysis and immunohistochemical (IHC) staining. Therefore, in this study, we supply evidence that the ALDOA-miR145-Oct4 axis can be used as a marker for cancer stem cells and as a new target for specific therapies.

## Materials and methods

### Cell lines

The human lung large cell type cancer cell lines H1299, H661, and H460 and the adenocarcinoma cell lines H1355, H1563, H441, CL1-0 and CL1-5 were cultured in RPMI 1640. All the culture media were supplemented with penicillin-streptomycin-glutamine (GIBCO; final concentrations: 100 units of penicillin, 100 µg streptomycin, and 0.292 mg glutamine per milliliter of medium) and FBS (Invitrogen). All cells were incubated in a 37 °C incubator under an atmosphere containing 5% CO_2_. The CL1-0 and CL1-5 cell lines were obtained from Chu and colleagues at the National Taiwan University, Taiwan. The other lung cancer cell lines were purchased from the American Type Culture Collection (ATCC).

### Sphere formation ability assay

The sphere formation medium was made by supplementing RPMI with 20 ng/ml of EGF, 20 ng/ml of bFGF and B27 for cancer cell in ultra-low attachment dishes. To generate spheres, 5 × 10^3^ cells were seeded into 6-well plates and incubated for 7–14 days in sphere formation medium. The spheres were then suspended in sphere formation medium and recounted for the 2^nd^ population of spheroids.

### Site-directed mutagenesis assay

The human *ALDOA* gene was cloned into the plenti6.3 lentiviral vector. Site-directed mutagenesis of the D33A, K293A and Y361S residues was performed using a GeneArt Site-Directed Mutagenesis kit (Thermo Fisher, Waltham, MA, USA) according to the manufacturer’s instructions.

### Cell viability assay

For lung cancer cells, 1000 cells were seeded in 96-well plates and treated with the indicated drugs for the indicated amounts of time. After 72 h, the cells were analyzed with alamarBlue dye according to the manufacturer’s instructions and were quantified using six replicates.

### Microarray analysi**s**

Total RNA extracted from cells with an A260/280 ratio greater than 1.9 was used in an Affymetrix cDNA microarray analysis. Hybridization was performed using Affymetrix human U133 2.0 plus arrays, and the chips were scanned on an Affymetrix GeneChip scanner 3000. Then, the Affymetrix DAT files were processed using the Affymetrix Gene Chip Operating System (GCOS) to generate CEL files. The raw intensities in the CEL files were normalized with a robust multichip analysis, and the fold-change analysis was performed using GeneSpring GX11 (Agilent Technologies).

### TMA immunohistochemistry staining assay

The tumor sections were prepared as formalin-fixed paraffin embedded tissues and were selected according to the morphology catalog associated with the diagnosis. We performed immunohistochemical staining on the tissue microarrays (TMA) using a Discovery XT automated immunostainer (Ventana Medical Systems, Tucson, AZ). The sections were dewaxed in an oven at 60 °C and deparaffinized with xylene followed by rehydration in an alcohol solution. Then, TRIS-EDTA buffer, with heat, was used to induce antigen retrieval. The antibodies used for immunostaining were as follows: ALDOA: Cat. T0891, 1:200 (Abcam (Epitomics), Cambridge, UK); Oct4: Cat. ab19857, 1:200 (Abcam, Cambridge, UK); TRAF4: Cat. 10083-2-AP, 1:200 (Proteintech, IL, USA); DUSP4: Cat. ab72593, 1:200 (Abcam, Cambridge, UK); and IgG: Cat. 7074, 1:200 (Cell Signaling, MA, USA). Subsequently, the tissue sections were counterstained with hematoxylin.

### Western blot analysis

The cells were lysed at 4 °C in RIPA buffer supplemented with protease and phosphatase inhibitors. Equal amounts of protein (30 μg) were electrophoretically separated using SDS-polyacrylamide gels and were then transferred to PVDF membranes (Millipore, Bedford, MA, USA). After blocking with 5% nonfat milk, the membranes were incubated overnight at 4 °C with the following specific antibodies: ALDOA: Cat. T0891, 1:1000 (Abcam (Epitomics), Cambridge, UK); ALDH1A3: Cat. GTX10784, 1:5000 (GeneTex, Taipei, Taiwan); Oct4: Cat. ab19857, 1:1000 (Abcam, Cambridge, UK); Nanog: Cat. 4903, 1:1000 (Cell Signaling, MA, USA); CD44: Cat. 3570, 1:1000 (Cell Signaling, MA, USA); BTK: Cat. sc-81159, 1:1000 (Santa Cruz, TX, USA); and Nestin: Cat. 66259-1, 1:1000 (Proteintech, IL, USA). Subsequently, the membranes were incubated with a horseradish peroxidase conjugated secondary antibody for 1 h. The blots were visualized using an ECL-Plus detection kit (PerkinElmer Life Sciences, Boston, MA, USA).

### Animal model experiments

All animal studies were performed in accordance with a protocol approved by the Academia Sinica Institutional Animal Care and Utilization Committee. Age-matched severe combined immune deficiency mutation and IL2 receptor gamma chain deficiency (NOD/SCID gamma) male mice (6–8 weeks old), originally from The Jackson Laboratory, were used in this study. To estimate in vivo tumor growth, 1 × 10^3^ and 1 × 10^4^ cancer stem cells were resuspended in 0.1 ml of PBS and injected into the left flank of mice (*n* = 5). Randomize mice for animal experiments in these manuscript.

### In silico database analysis

The clinical information and genomic matrix file of The Cancer Genome Atlas (TCGA) database were download from the USCS Xena browser website (https://xenabrowser.net/heatmap/). All GSE serious datasets were downloaded from the Gene Express Omnibus (GEO) website and were normalized and analyzed via Genspring software (Version 13.1.1, Agilent, Santa Clara, CA, USA). All of the data downloaded included the clinical parameters and expression level of the target genes in lung patients from the Xena browser. This website applied microarray or next-generation sequencing of each probe after normalization, and for high expression, the median was set higher than that for high expression, and vice versa. In addition, we removed several clinical cases that lacked the corresponding parameters.

### Quantitative reverse-transcription polymerase chain reaction assay

Total RNA was extracted with Trizol, and RNA (1 μg) was added as a template to RT reactions performed with a SuperScript III kit (Invitrogen, Carlsbad, CA, USA). Data for the RT-qPCR were normalized to the GAPDH gene. RT-qPCR was conducted with the obtained complementary (c)DNA9 in triplicate using TaqMan Universal Master Mix II (Applied Biosystems, Foster City, CA, USA), primers and probes that were purchased from Applied Biosystems (cat. no.: Hs00855401_g1, Applied Biosystems), and an ABI 7000 real-time PCR machine (cat. no.: Hs00258287_m1, ABI, Foster city, CA, USA) was used. The experimental Ct values were normalized to that of GAPDH.

### Statistical analysis

The non-parametric Mann-Whitney *U*-test was used to analyze the statistical significance of results from three independent experiments. Statistical analyses were performed using SPSS (Statistical Package for the Social Sciences) 17.0 software (SPSS, Chicago, IL, USA). The association between IC50 of chemotherapeutic drugs and the ALDOA expression levels were analyzed by Pearson’s chi-square test. Estimates of the survival rates were calculated using the Kaplan-Meier formula and compared using the log-rank test. Follow-up time was censored if the patient was lost during follow-up. Univariate and multivariate analyses were performed using Cox proportional hazards regression analysis with and without an adjustment for ALDOA and other candidate targets expression level, grade. For all analyses, a *P*-value of <0.05 was considered significant.

## Results

### Aldolase A is associated with chemoresistance in lung cancer cells

**Fig. 1:**
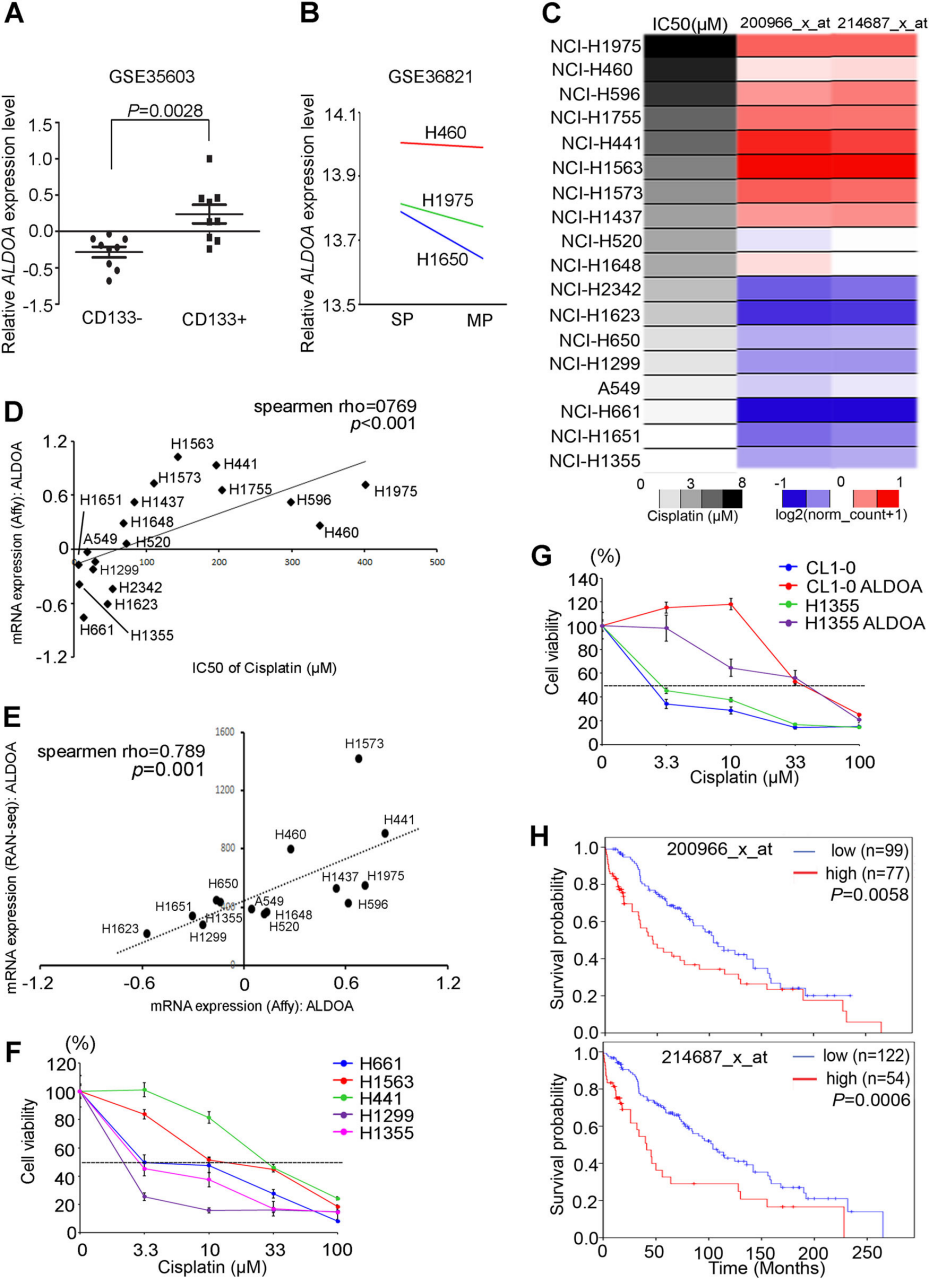
ALDOA is positively correlated with chemoresistance to cisplatin in lung cancer cells. **a** Box plot showing the *ALDOA* expression levels in lung tumor stem-like cells from the GSE35603 dataset indicating positive/negative expression of CD133. **b** The *ALDOA* gene expression data were obtained from the GSE36821 database for parental cells and stem-like cells in several lung cancer cell lines (MP: primary population, SP: side population). **c** Heat map showing the endogenous level of *ALDOA* mRNA expression and the IC50 value of cisplatin treatment in lung cancer cells from in silico datasets. **d** Correlation between the *ALDOA* mRNA level and the IC50 value of cisplatin treatment of various lung cancer cell lines from the COSMIC website. **e** Scatter plot showing the correlation between the *ALDOA* mRNA level in the microarray and *ALDOA* RPKM in the RNA-Seq analysis. **f** The alamarBlue assay was used to measure cell viability in the various lung cancer cell lines, including H661, H1563, H441, H1299 and H1355 cells, which were treated with cisplatin (0, 3.3, 10, 33 and 100 μM). **g** The alamarBlue assay was used to measure cell viability of CL1-0 and H1355 cells, with or without the overexpression of an exogenous ALDOA gene, treated with cisplatin (0, 3.3, 10, 33 and 100 μM). **h** Kaplan-Meier analysis of *ALDOA* expression at concurrently low or high levels for the endpoint of overall survival probability of lung cancer patients after chemotherapy treatment obtained from the Kaplan-Meier Plotter database (*n* = 176).

Drug response was recently revealed to be an important issue in clinical treatment. However, a number of patients still exhibit resistance and recurrence events after drug treatment. Cancer stem cells form spheroids to avoid drug attack and allow recurrence. In addition, metabolic reprogramming has been shown to be important for cancer stemness. However, the detailed mechanism and the ratio between glycolysis and OXPHOS remain unknown. To solve this problem, we proposed that glycolysis-associated genes may trigger the metabolic reprogramming of sphere formation and drug resistance in lung cancer. Therefore, we established microarray chips using the highly sphere-forming cell line CL1-5 and its counterpart cell line CL1-0. The data showed that the gene expression of several glycolytic enzymes was causally associated with the sphere formation ability of human lung adenocarcinoma. We analyzed the expression of glycolysis-associated genes from several in silico studies, with our results demonstrating that ALDOA plays a unique role and correlates with lung cancer stemness. An increase in the expression of ALDOA in CD133-positive lung cancer cells or a side population of cells compared with that of the control groups was observed (Fig. 1a, b). Furthermore, we collected data on the mRNA expression level for each lung cancer cell line from the CCLE (GSE36133) and the RNA-Seq cohorts. We also obtained the corresponding IC50 values for several chemotherapy drugs from the COSMIC and CCLE databases. Via data searches and manipulation, we observed that the ALDOA RNA levels were positively correlated with the IC50 values of chemotherapy drugs such as cisplatin in a lung cancer cell panel according to two independent analyses (Fig. 1c). These data also showed that the level of ALDOA RNA was strongly correlated with the IC50 value of cisplatin for both the COSMIC and CCLE cohorts (Fig. 1d and Supplementary Fig. S1). We further verified that the trend was consistent between the RPKM and the peak score by evaluating RNA-Seq data from the CCLE cohort. The results also showed a positive correlation between the RNA-Seq and CCLE datasets (Fig. 1e). Therefore, we assessed the cell viability of several lung cancer cell lines and ALDOA overexpression models via an MTT assay. We observed that the level of ALDOA expression was indeed associated with the IC50 value of cisplatin (Fig. 1f). Furthermore, our data showed that ALDOA enhanced drug resistance in sensitive lung cancer cells, including CL1-0 and H1355 cells (Fig. 1g). In addition, we observed that the level of ALDOA expression was correlated with poor survival in a chemotherapy treatment specific cohort using two independent probes (Fig. 1h). ALDOA not only responds to cisplatin in lung cancer cell lines but our observations also identified several standard chemotherapeutic drugs associated with ALDOA expression in lung cancer, including paclitaxel and vinorelbine (Supplementary Table 1). Nevertheless, the IC50 value of cisplatin displayed the most significant correlation with the ALDOA expression level. Considering these results, we hypothesized that ALDOA combats the effects of chemotherapy drugs in lung cancer.

### ALDOA enhances the spheroid formation ability in vitro and initiation ability in vivo

**Fig. 2:**
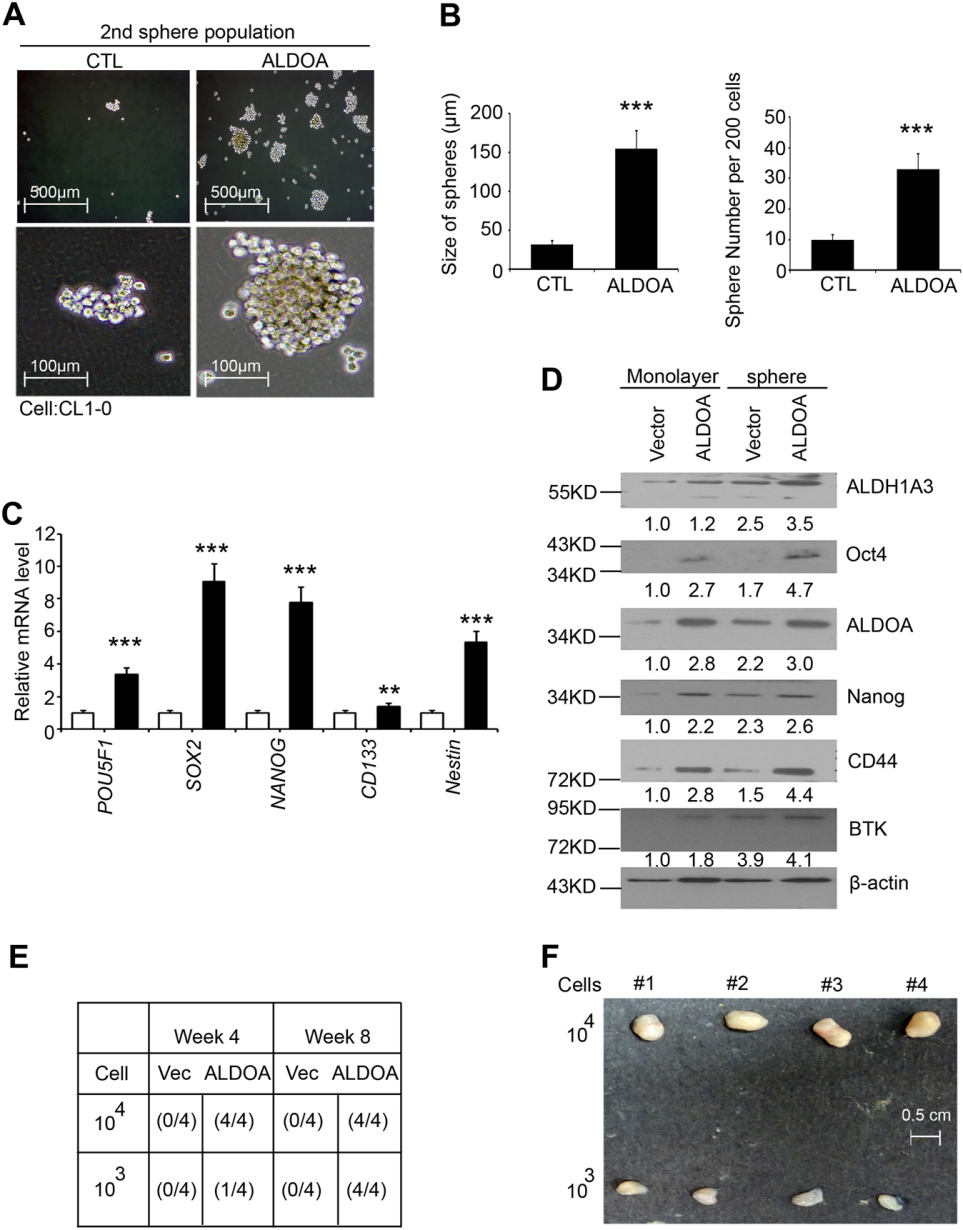
ALDOA determines the cancer properties and self-renewal capability of lung cancer. **a** Representative images of the density and morphology of spheroids at various magnifications using a 2^nd^ population of the vector control and ALDOA expression construct in CL1-0 cells. **b** Quantitative analysis results of the spheroid numbers and diameters after control and ALDOA overexpression treatment are shown in the panels. **c** The qRT-PCR results show the mRNA expression of *POU5F1*, *SOX2*, *NANOG*, *CD133* and *Nestin* with or without overexpression of an exogenous ALDOA gene in CL1-0 cells. **d** Western blot analysis showing the protein levels of ALDH1A3, Oct4, ALDOA, Nanog, CD44 and BTK with or without overexpression of an exogenous ALDOA-encoding gene in CL1-0 monolayer and sphere-forming cells. **e**, **f** An overview of the appearance of mice implanted with ALDOA-overexpressing CL1-0 sphere-forming cells by a subcutaneous injection in the right flank using serially diluted cells. In **c**, *GAPDH* was used as an internal loading control. The significance of the differences in **b** and **c** was analyzed using Student’s *t*-test.

From the transcriptomics datasets obtained from the ALDOA-induced cell models, we did not observe significant changes in resistance-related signatures, such as for the ATP-binding cassette subfamily B member 1 (ABCB1) and P-glycoprotein. Therefore, we hypothesized that ALDOA may contribute to the formation of spheres and further protect against chemotherapeutic drugs. To assess whether spheroid formation was induced by ALDOA expression, we established primary and secondary spheroid cultures from ALDOA overexpression models by lentiviral infection (Fig. 2a and Supplementary Fig. S2). The results showed a higher spheroid size and number (per 200 cells) in the overexpression group compared with those in the vector control (Fig. 2b). The qRT-PCR results confirmed that well-known CSC markers, such as *POU5F1*, *SOX2*, *Nanog*, *CD133* and *Nestin*, were expressed at higher levels in the CSC-like cells when ALDOA was induced (Fig. 2c). The Western blot results also showed that the CSC-related markers were upregulated in sphere-forming cells compared with the majority of parental cells (Fig. 2d).

We evaluated whether ALDOA also modulates the tumor-initiating potential of lung CSCs. Primary lung cancer cells were overexpressed with an empty control or an ALDOA exogenous plasmid, and secondary spheroids were generated. These groups were tested in tumor initiation assays through a subcutaneous injection of serially diluted cells (10^3^ and 10^4^ cells in 100 µl) into immune-deficient mice. Four weeks after the injection, ALDOA-expressing cells formed tumors in 1 out of 4 mice injected with 10^3^ cells in 100 µl and in all of 4 mice injected with 10^4^ cells in 100 µl, with tumor latencies of 20 and 40 days, respectively. In contrast, no tumors were observed in mice injected with the empty control-expressing cells. At 8 weeks, all mice injected with the empty control-expressing cells remained tumor-free, while tumors were detected in all four mice bearing the ALDOA-overexpressing tumor cells (Fig. 2e). Our data also revealed that ALDOA triggered stemness in lung cancer cells, with the spheroids exhibiting initiation and growth abilities in vivo (Fig. 2f).

**Fig. 3:**
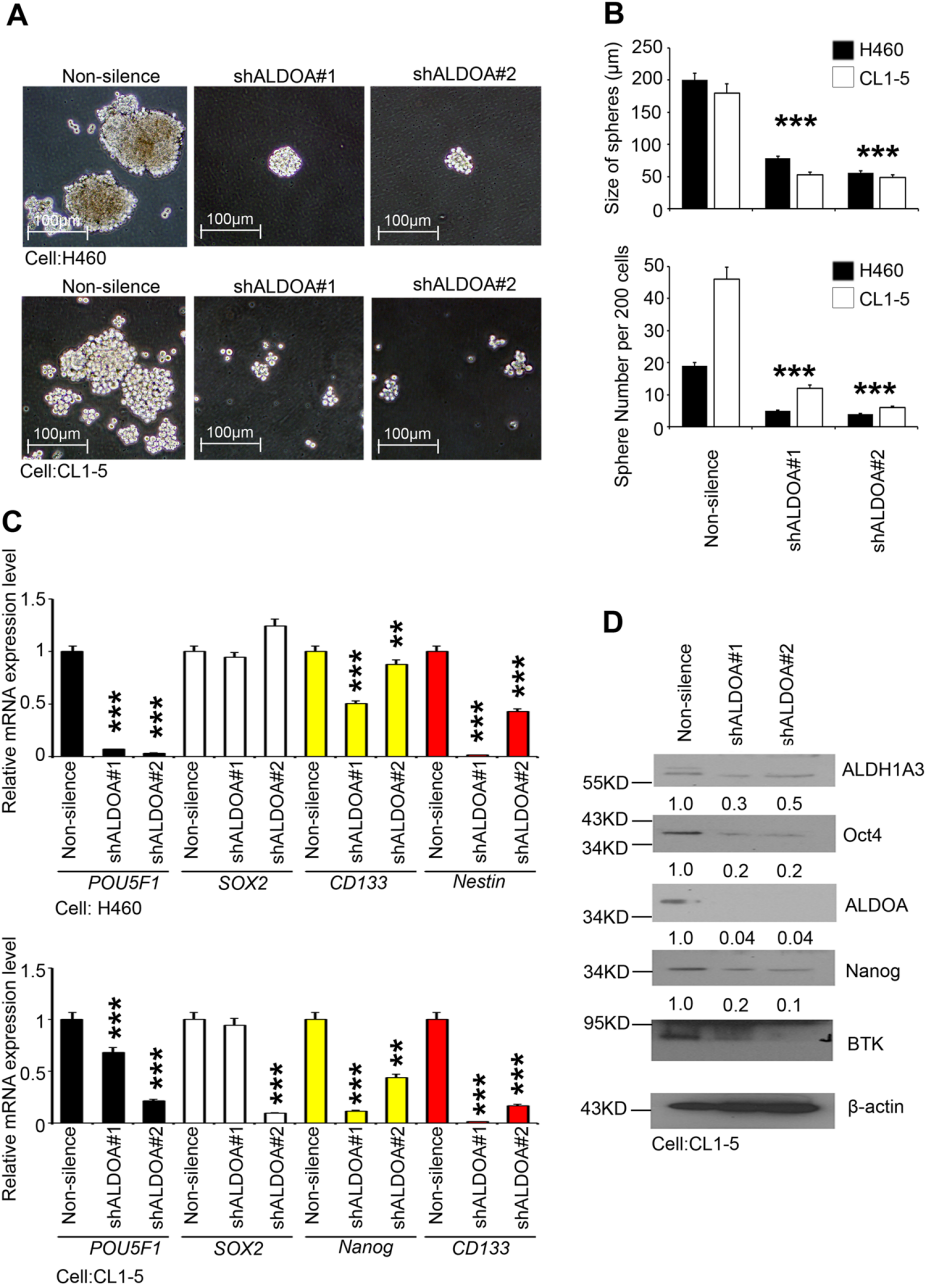
ALDOA expression inhibits lung cancer spheroid formation. **a** Representative images of the density and morphology of the spheroids in the nonsilenced group and with ALDOA knockdown in H460 and CL1-5 cells. **b** Quantitative analyses of the spheroid number and diameter in the nonsilenced and ALDOA knockdown groups are shown in the panels. **c** qRT-PCR results showing the mRNA expression of *POU5F1*, *SOX2*, *NANOG* and *CD133* and *Nestin* in the nonsilenced group and with ALDOA knockdown in H460 and CL1-5 cells. **d** Western blot analysis of the protein levels of ALDH1A3, Oct4, ALDOA, Nanog and BTK in the nonsilenced group and with ALDOA knockdown in CL1-5 cells. The significance of the differences in **b** and **c** was analyzed using Student’s *t*-test.

In addition, we assessed the impact of ablating ALDOA expression using short hairpin RNAs (shRNAs) in the high spheroid-formation cell lines CL1-5 and H460 (Fig. 3a). The silencing of ALDOA with two independent shRNAs resulted in a decrease in both the spheroid size and the number per 200 cells (Fig. 3b). The qRT-PCR and Western blot results showed that several CSC markers were suppressed by the ALDOA shRNAs (Fig. 3c, d). Taken together, these results demonstrate that ALDOA plays a key role in lung cancer stemness and tumor initiation.

### ALDOA induces cancer stemness through a nonenzymatic mechanism

**Fig. 4:**
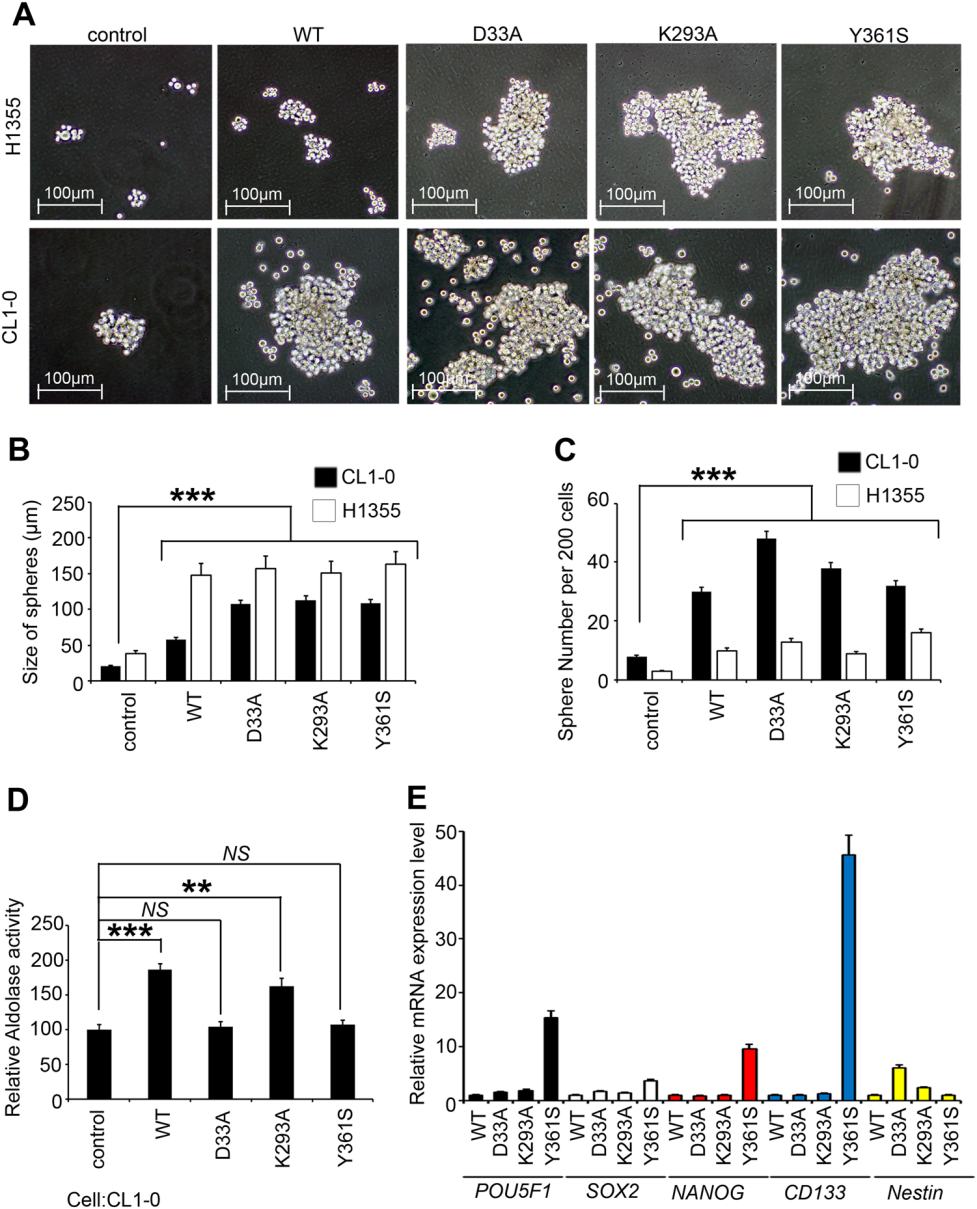
ALDOA promotes cancer stemness ability through a nonenzymatic mechanism. **a** Representative images of the density and morphology of spheroids from CL1-0 and H1355 cells expressing the vector control, wild-type and several mutant forms of ALDOA. **b** Quantitative analysis of the spheroid **b** diameters and **c** numbers in the control and in several ALDOA overexpression models is shown on the panels. **d** Intracellular aldolase activity in the control group and in several ALDOA overexpression models. **e** qRT-PCR results showing mRNA expression of *POU5F1*, *SOX2*, *NANOG*, *CD133* and *Nestin* with or without overexpression of an exogenous ALDOA-encoding gene in CL1-0 cells. In **e**, *GAPDH* was used as an internal loading control. The significance of the differences in **b**–**e** was analyzed using Student’s *t*-test.

The results of previous studies have revealed that sphere formation or cancer stemness may be correlated with the Warburg effect or further metabolic reprogramming^19–21^. Additional observations have indicated that lung and GBM cancer stem cells may contribute to the formation of spheroids in OXPHOS, whereas breast cancer, colon cancer and liver cancer stem cells preferentially use a glycolytic shift^21,22^. To determine whether the ability of ALDOA to form spheroids was induced by glycolysis, OXPHOS or even an enzymatic-independent mechanism, we screened several metabolic events between attachment and spheroid formation in cultured lung cancer cells. We consistently observed that lung cancer stem cells induced metabolic reprogramming from the glycolytic to the OXPHOS pathways rather than due to parental attachment types. Moreover, we generated several mutant forms of ALDOA with abrogated enzymatic activity for further experiments^23–25^. Initially, we created several mutant forms of ALDOA with substitutions in crucial catalytic site residues, including D33A, Y361S, and K293A, for use as negative controls in this experiment (Fig. 4a). We observed that the wild-type and mutant forms of ALDOA promoted cancer stemness. We calculated the size of the spheroids and the sphere number per 200 cells, with the results showing that the wildtype and all the mutants increased the sphere formation ability compared with that of the control group (Fig. 4b, c). Furthermore, we validated the enzyme activity of ALDOA using the wildtype and several mutants. The enzymatic activity of ALDOA was significantly decreased in the D33A and Y361S mutants but not the wild-type and K293A enzymes (Fig. 4d). These results revealed a trend consistent with those previously observed for wild-type ALDOA (Figs. 2a and 4d). Moreover, we evaluated the expression levels of CSC makers in these cell models via qRT-PCR analysis. We validated that ALDOA indeed promotes lung cancer cell spheroid formation in an enzyme activity-independent manner (Fig. 4e).

### ALDOA regulates Oct4 activity by suppressing miR-145

**Fig. 5:**
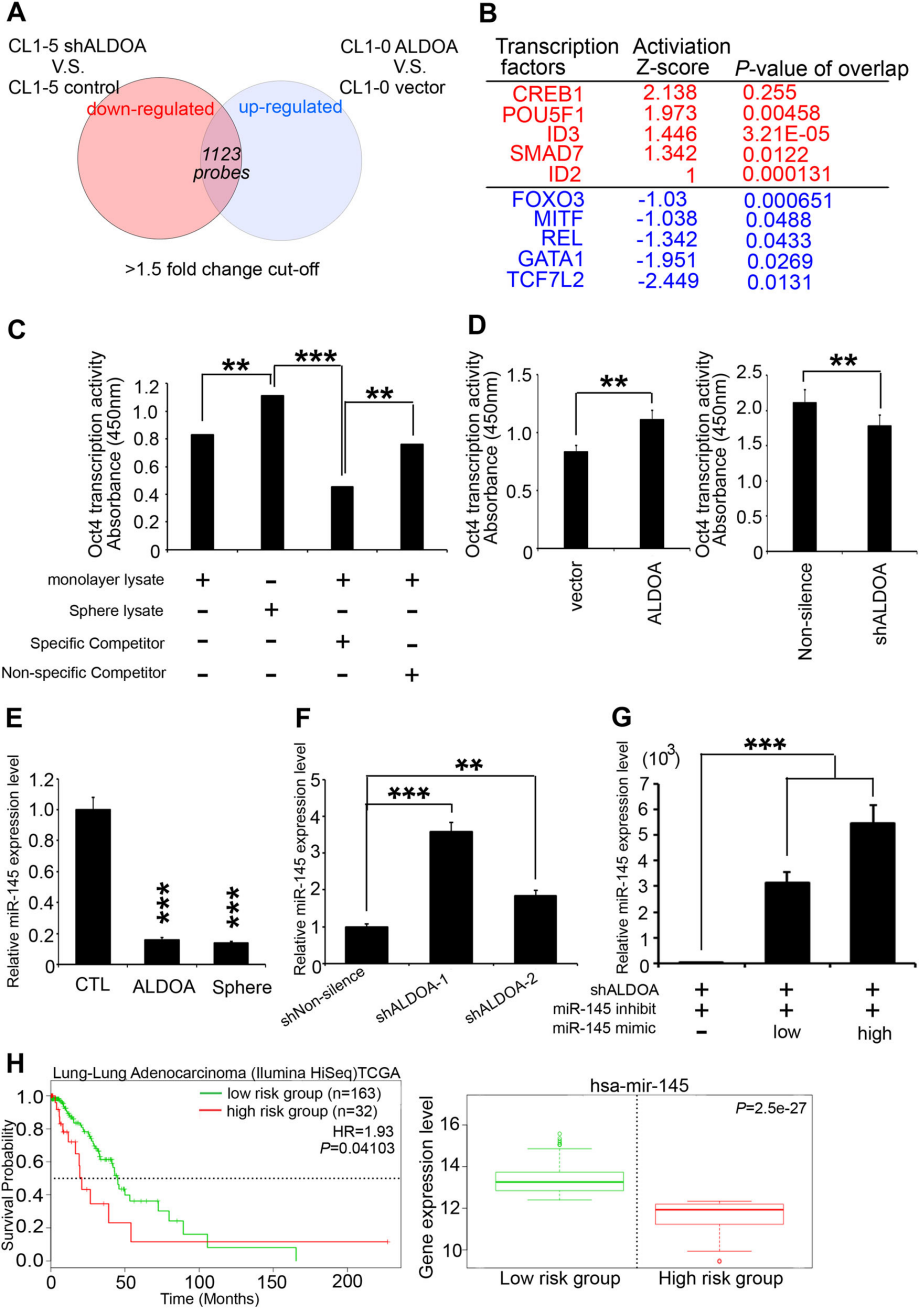
ALDOA triggers Oct4 transcriptional activity via miR-145 suppression. **a** Venn diagram of the overlap among the genes upregulated by ALDOA versus the control in the CL1-0 cells and the downregulated genes in shALDOA versus the shnon-silenced CL1-5 cells. The genes up/downregulated by more than 1.5-fold in the cells and those associated with unfavorable prognosis were selected using microarrays and survival data analysis. **b** Ranking of the candidate upstream transcriptional regulators and the corresponding z-score. *P*-value obtained using the IPA database from a microarray experiment performed with a common ALDOA signature with a 1.5-fold change cut-off. **c** Intracellular Oct4 activity of cancer spheres and parental cells with and without a competitor treatment. **d** Intracellular Oct4 activity in an ALDOA two-way cell model. **e** qRT-PCR results showing the expression of miR-145 in the ALDOA overexpression model or cancer spheres in CL1-0 cells. **f** qRT-PCR results showing the expression of miR-145 with shnon-silenced or shALDOA knockdown clones in the CL1-5 shALDOA models. **g** qRT-PCR results showing the expression of miR-145 in the ALDOA knockdown model with or without the miR-145 mimic combined with miR-145 inhibitor treatment in CL1-5 cells. **h** Kaplan-Meier analysis of has-mir-145 expression at concurrently low or high levels (or other levels) with the endpoint of overall survival probability of lung cancer patients obtained from the Kaplan-Meier Plotter database (*n* = 195). In **e**–**g**, *GAPDH* was used as an internal loading control. The significance of the differences in **c**–**g** was analyzed using Student’s *t*-test.

To gain additional insights into the molecular mechanisms involved in ALDOA-induced sphere formation, we established ALDOA two-way microarray chips using Genespring software for normalization and selected the probes with a >1.5-fold change for prediction analysis. We performed ingenuity pathway analysis (IPA)-upstream regulator analysis using the 1,123 gene set (Fig. 5a). We identified *POU5F1*, also known as Oct4, as a significant upstream transcriptional regulator in our model system (Fig. 5b) and subsequently analyzed its transcriptional activity in our models. We observed that the transcriptional activity of Oct4 was upregulated in sphere-forming cells and cells stably overexpressing ALDOA. In contrast, a specific competitor of Oct4 and an ALDOA knockdown model suppressed Oct4 transcription (Fig. 5c, d). Importantly, to date, no study has demonstrated that ALDOA can regulate the activity of transcription factors, and we hypothesized that microRNAs regulate the transcriptional activity of Oct4 in our available sphere-forming cells. Moreover, selected pseudogenes act as microRNA decoys to regulate the effects of microRNAs on the corresponding protein. The use of a website for microRNA predictions showed that several candidate microRNAs may potentially target Oct4 (Supplementary Table 2). We further determined the effects of the expression levels of several microRNAs on survival and validated miR-145 as the most important regulator of Oct4 in our model (Supplementary Figs. S3 and S4a)^26,27^. Using a qRT-PCR assay, we also observed that miR-145 is downregulated in ALDOA-overexpressing cells (Fig. 5e, f). Therefore, we recruited miR-145 inhibitor to validate the increased miR-145 expression levels in ALDOA knockdown models. We also added a miR-145 mimic in the available model, and we observed that the expression levels of miR-145 were partially restored in the ALDOA knockdown models (Fig. 5g). Additionally, our results showed that the miR-145 expression level was suppressed in ALDOA overexpressing cells. (Supplementary Fig. S4b). In the clinical component, we also found that miR-145 expression was correlated with patient survival (Fig. 5h) in lung cancer. Based on these results, we proposed a putative model in which ALDOA triggers Oct4 transcriptional activity through the downregulation of miR-145, which explains why miR-145 could not promote Oct4 degradation in our model.

### ALDOA promotes cancer stemness through the Oct4-DUSP4/TRAF4 axis

**Fig. 6:**
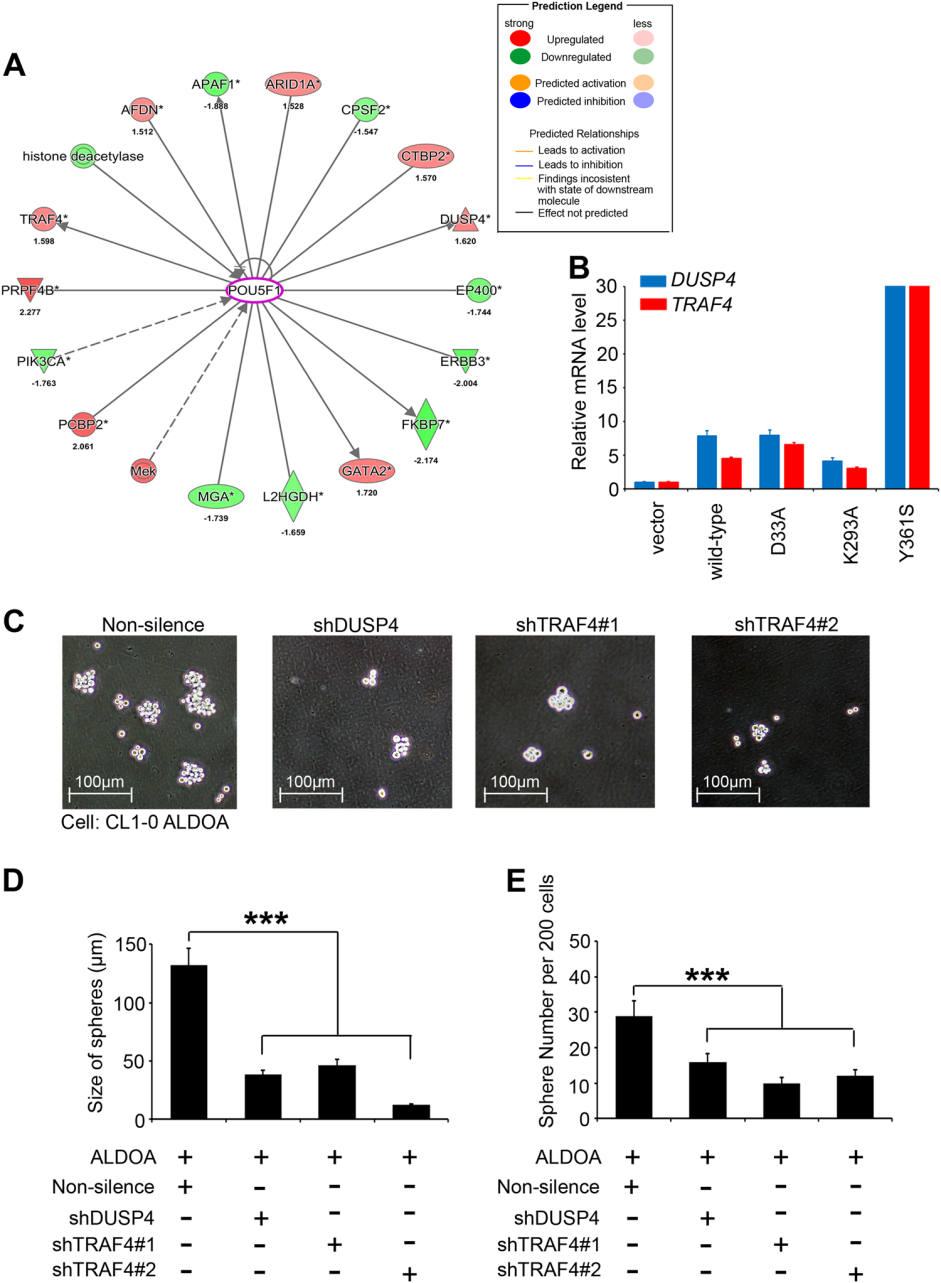
ALDOA regulates stemness properties in lung cancer via the Oct4-DUSP4/TRAF4 axis. **a** Core analysis was performed based on the selection criteria in the IPA database overlaid with the microarray data from the ALDOA common signature using a 1.5-fold change cut-off. **b** qRT-PCR results showing the mRNA expression of *DUSP4* and *TRAF4* with or without overexpression of an exogenous ALDOA-encoding gene in CL1-0 cells. **c** Representative images of the density and morphology of spheroids in the nonsilenced group and with DUSP4 and TRAF4 knockdown in CL1-0 ALDOA-overexpressing cells. **d** Quantitative analysis of the spheroid diameters for the nonsilenced group and for those with several downstream targets that were knocked down in CL1-0 ALDOA cells is shown in the panels. **e** Quantitative analysis of the spheroid numbers per 200 cells for the nonsilenced group and for those with several downstream targets that were knocked down in the CL1-0 ALDOA cells is shown in the panels. In **b**, *GAPDH* was used as an internal loading control. The significance of the differences in **b** and **d** was analyzed using Student’s *t*-test.

Combining several lines of evidence, we showed that ALDOA promotes lung cancer cell stemness through a nonenzymatic mechanism. Moreover, we wanted to elucidate which downstream factors play important roles in the ALDOA-Oct4 axis. Using IPA software prediction analysis, we observed that Oct4 activation may upregulate the level of *DUSP4* and *TRAF4* expression (Figs. 6a and Supplementary Fig. S5). In contrast, Oct4 may downregulate the level of *APAF1* and *PPARA* expression (Fig. 6a). We further screened the RNA levels of several candidate genes and validated the expression status of DUSP4 and TRAF4 in our stable cell models. Our data were consistent with previous microarray chip results for both wild-type and multiple mutant forms of ALDOA (Fig. 6b). Via knockdown of each gene using shRNAs, we observed that DUSP4 or TRAF4 alleviated the phenotype induced by ALDOA (Fig. 6c–e). Thus, ALDOA regulates the stemness properties of lung cancer cells via the Oct4-DUSP4/TRAF4 axis.

### Clinical significance of the ALDOA and Oct4 axis in Lung CSC

**Fig. 7:**
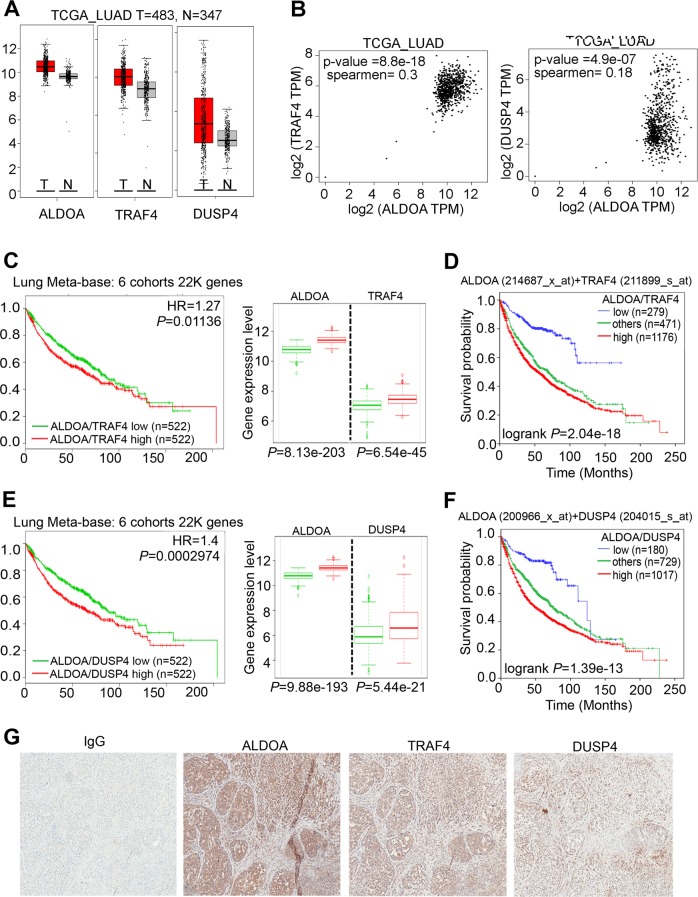
ALDOA coordinates with DUSP4/TRAF4 and correlates with a poor prognosis in lung cancer patients. **a** Box-plots showing the endogenous mRNA expression levels of ALDOA, DUSP4 and TRAF4 in the tumor or adjacent normal tissues of lung cancer patients from the TCGA clinical cohort. **b** Correlation plots between ALDOA with the TRAF4 or DUSP4 expression levels in the TCGA_LUAD clinical cohort. **c** Kaplan-Meier analysis of TRAF4 combined with ALDOA gene expression, as identified in the meta-base cohort (Lung Meta-base: 6 cohorts 22k genes, *n* = 1044) from the Survexpress website with the endpoint of overall survival. **d** Kaplan-Meier analysis of ALDOA (probe ID = 214687_x_at) combined with TRAF4 (probe ID = 211899_s_at) gene expression at concurrently low or high levels (or other levels) with the endpoint of overall survival probability of lung cancer patients obtained from the Kaplan-Meier Plotter database (*n* = 1926). **e** Kaplan-Meier analysis of DUSP4 combined with ALDOA gene expression, as identified in the meta-base cohort (Lung Meta-base: 6 cohorts 22k genes, *n* = 1044) from the Survexpress website, with the endpoint of overall survival. **f** Kaplan-Meier analysis of ALDOA (probe ID = 200966_x_at) combined with DUSP4 (probe ID = 204015_s_at) gene expression at concurrently low or high levels (or other levels) with the endpoint of overall survival probability of lung cancer patients obtained from the Kaplan-Meier Plotter database (*n* = 1926). **g** IHC staining of the negative control IgG and ALDOA, Oct4, DUSP4 and TRAF4 in mice implanted with ALDOA-overexpressing CL1-0 spheroids via subcutaneous injection in the right flank.

**Fig. 8:**
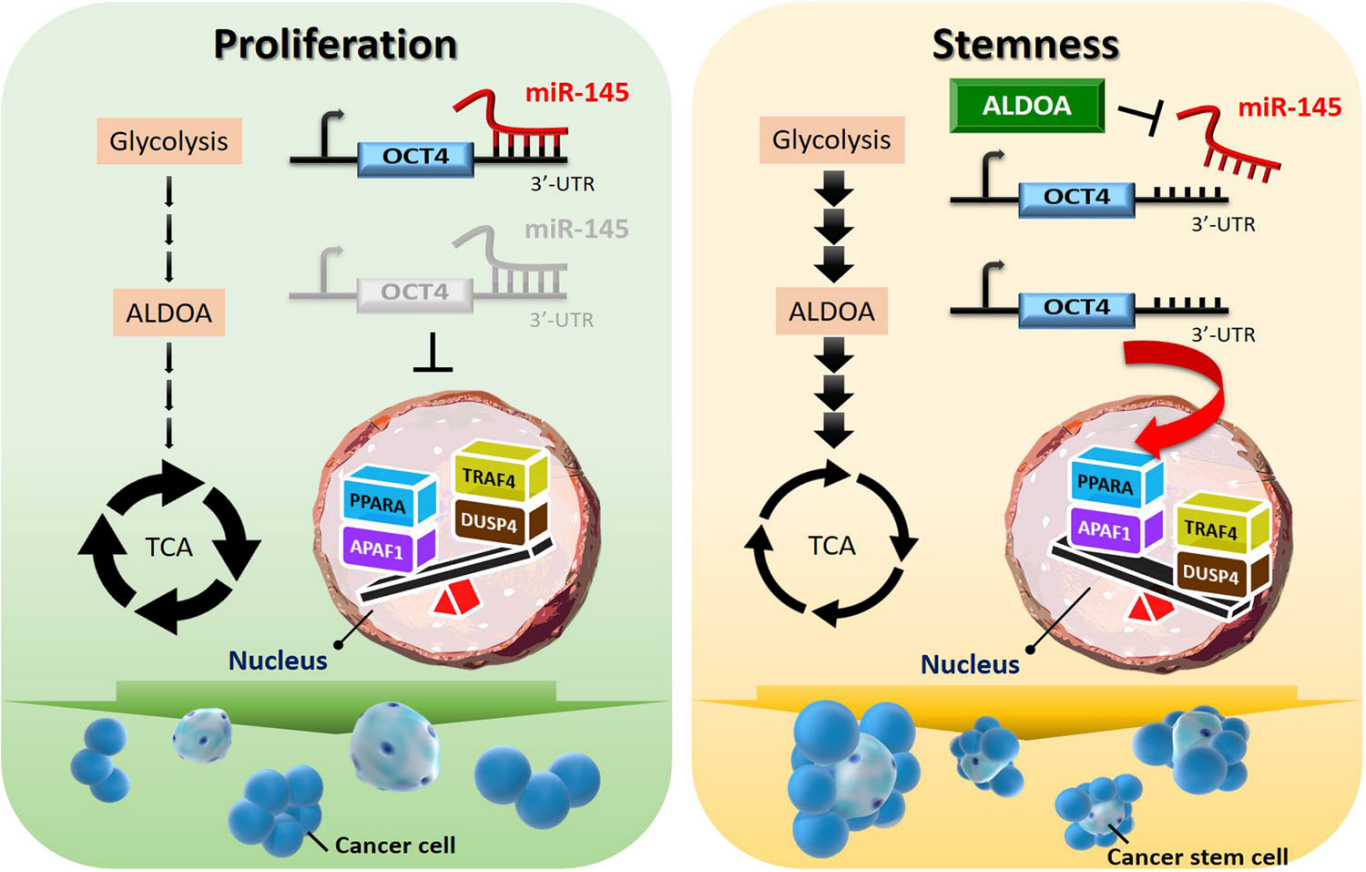
Schematic model of the ALDOA-Oct4 axis in lung cancer progression. The proposed model illustrates the increased Oct4-mediated stemness by ALDOA in spheroid formation and activation of downstream TRAF4/DUSP4 activity.

To elucidate the ALDOA-Oct4-TRAF4/DUSP4 axis, we assessed whether RNA and proteins were expressed at different levels between primary tumors (*n* = 971) and their adjacent normal tissue (*n* = 153). At the RNA level, we observed that ALDOA coordinates with TRAF4 or DUSP4 in a TCGA lung cancer patient cohort. All the target genes were upregulated in tumors compared with normal tissues (Fig. 7a and Supplementary Fig. S6). In addition, we verified the positive correlation between ALDOA expression and that of TRAF4 and DUSP4 in the TCGA cohort (Fig. 7b). Furthermore, by combining those genes into a significant prognostic factor for lung cancer patients, we determined that ALDOA, together with TRAF4 or DUSP4, serves as an independent factor for the meta-base and TCGA lung cancer patient cohorts (Fig. 7c–f). Moreover, we dissected solid tumors from lung CSC xenograft models and determined the protein levels of several candidate targets by IHC staining, which revealed a trend similar to that observed for the RNA levels (Fig. 7g). Finally, we demonstrated a model in which ALDOA coordinates with DUSP4/TRAF4 and is correlated with a poor prognosis in lung cancer patients (Fig. 8).

## Discussion

An increased rate of glycolysis or metabolic reprogramming is viewed as a hallmark of cancer and has been implicated in other diseases^28^. Glycolysis plays an initial role in cellular metabolism and regulates downstream pathways through the formation of intermediates and products^29^. The aldolase family of proteins has many important functions in cell biology. Aldolase controls glycolysis, metabolic events and downstream amino acid/lipid/energy synthesis. Furthermore, aldolase dysfunction has been reported in multiple cancer types^30^. However, the nonenzymatic function of aldolase family members remains unknown, with conflicting opinions expressed on the subject. In a previous study, increased ALDOA enzyme activity was observed during tumorigenesis, further enhancing lactate production and metabolic reprogramming. In addition, several studies have shown that ALDOA regulates channel pumps^31^, the cell cytoskeleton^32^ and signal transduction through protein–protein interactions. Interestingly, in this study, we demonstrated that ALDOA also has a novel nonenzymatic function in promoting cancer stemness.

Because ALDOA has been shown to be associated with the IC50 values of chemotherapeutic drugs in lung cancer cells, we hypothesized that ALDOA is involved in lung cancer stemness and investigated the correlation between ALDOA and *POU5F1* (protein name: Oct4), one of the most important regulators of cancer stemness. We showed that miR-145, a microRNA that directly binds *POU5F1*, was suppressed by ALDOA. Because ALDOA is a member of the glycolytic family of enzymes, we generated several mutants of ALDOA in the catalytic region to block its enzymatic activity, including D33A and Y361S substitutions. We showed that with or without enzymatic activity, ALDOA retained its ability to activate *POU5F1* and its downstream factors *DUSP4* and *TRAF4* through miR-145 suppression, although the details of the associated mechanism of action remain unclear. However, this axis may potentially be a potential target for drug development and therapy against cancer stemness and the prevention of drug resistance. In the future, we plan to knock out ALDOA through a CRISPR approach to verify that aldolase has been completely blocked. We anticipate that sphere formation will not be inhibited in the ALDOA enzyme deprivation models or will be significantly increased by overexpressing ALDOB or ALDOC.

Tumor necrosis factor receptor-associated factor 4 (TRAF4) belongs to the TRAF family, members of which are associated with and mediate signal transduction from members of the TNF receptor superfamily^33^. An observed overexpression of TRAF4 suggested that it is associated with the initiation and progression of primary breast cancer and metastasis^34^. Moreover, several lines of evidence indicate that TRAF4 regulates embryogenesis and central nervous system merlin homeostasis^35^. Gao et al. reported that TRAF4 is involved in stemness in esophageal squamous cell carcinoma (ESCC) and also validated that has-miR-21-3p directly regulates TRAF4 to promote the proliferation and antiapoptotic ability of ESCC-isolated stem cells^36^. Furthermore, dual specificity phosphatase 4 (DUSP4) has been reported to negatively regulate members of the MAP kinase superfamily, including MAPK/ERK, JNK and p38^37,38^. In addition, DUSP4 can promote epithelial-mesenchymal transition (EMT) and alter gastric cell morphology to increase resistance to doxorubicin^39,40^. Moreover, PKC-θ directly regulates the complex, including DUSP4 and p300, to maintain H3K27ac status during the EMT procedure^39^. Although these target genes are overexpressed in various human malignancies, the mechanism related to the roles of TRAF4 and DUSP4 in tumorigenesis remains unclear. However, several studies still consider DUSP4 to be one of the targets for treatment options in cancer research^41^. In this study, we demonstrated that TRAF4 and DUSP4 are directly upregulated by Oct4 binding to promote sphere formation and further drug resistance. Taken together, these results demonstrate a novel role of TRAF4/DUSP4 in stemness and drug resistance in lung carcinogenesis.

In this study, we showed that the glycolysis enzyme aldolase A (ALDOA) is correlated with the IC50 values of chemotherapy drugs in lung cancer cell lines. We further demonstrated that ALDOA triggers Oct4 activity and the downstream factors TRAF4/DUSP4 to promote spheroid formation and drug resistance in lung CSCs in an enzyme activity-dependent manner. Moreover, we verified that ALDOA also inhibits miR-145 expression to disrupt the direct binding of miR-145 to the 3′UTR region of Oct4. Clinical data from several available cohorts, including TCGA and meta-base 1000 patients with lung cancer, indicated that the ALDOA-Oct4-TRAF4/DUSP4 axis is a potential prognostic indicator and may be a therapeutic target. In non-small cell lung cancer, scientists are developing various strategies and methods for targeting and specificity^42,43^. This finding has strong potential for the development of novel cancer therapies or combination therapies against CSCs in lung cancer.

## Supplementary information

Supplement Table 1

Supplement Table 2

Supplement Figure legends

Supplement Figure 1

Supplement Figure 2

Supplement Figure 3

Supplement Figure 4

Supplement Figure 5

Supplement Figure 6
