# Supplementary material for: Nonenzymatic function of Aldolase A downregulates miR-145 to promote the Oct4/DUSP4/TRAF4 axis and the acquisition of lung cancer stemness
Source: Cell Death Dis. 2020 Mar 18;11(3):195. doi: 10.1038/s41419-020-2387-2 (PMC7080828; doi:10.1038/s41419-020-2387-2)
Supplement: Supplementary file 1 — Supplement Table 1 [file 41419_2020_2387_MOESM1_ESM.docx]

**Supplementary Table 1.** IC50 values of Paclitaxel and Vinorelbine in lung cancer cell lines.

|  | **200966_x_at** | **214687_x_at** | **Paclitaxel** | **Vinorelbine** |
| --- | --- | --- | --- | --- |
| **Cell** | **ALDOA** | **ALDOA** | **Dose(uM)** | **Dose(uM)** |
| **NCI-H1563** | 1.0257 | 1.0388 | 8 | 0.01 |
| **NCI-H1648** | 0.2865 | 0.1356 | 8 | 0 |
| **NCI-H441** | 0.9313 | 0.8314 | 0.13 | 0.03 |
| **NCI-H460** | 0.2675 | 0.2742 | 0.029 | 0.01 |
| **SK-LU-1** | 0.0119 | 0.0588 | 0.022 | N/A |
| **NCI-H1651** | -0.3859 | -0.3023 | 0.022 | 0.06 |
| **NCI-H2009** | 0.3851 | 0.3339 | 0.018 | 0.01 |
| **NCI-H1355** | -0.1755 | -0.1633 | 0.018 | 0 |
| **NCI-H1573** | 0.7294 | 0.6750 | 0.014 | N/A |
| **NCI-H1299** | -0.2254 | -0.2407 | 0.014 | 0.01 |
| **NCI-H520** | 0.0618 | 0.1221 | 0.007 | 0 |
| **NCI-H661** | -0.7599 | -0.7449 | 0.006 | 0.02 |
| **PC-14** | 0.3775 | 0.4011 | 0.004 | 0 |
| **NCI-H2122** | 0.4319 | 0.3994 | 0.003 | 0 |
| **NCI-H1975** | 0.7163 | 0.7126 | 0.0026 | N/A |
| **NCI-H650** | -0.1377 | -0.1407 | 0.002 | 0.02 |
| **NCI-H2228** | 0.1776 | 0.1778 | 0.002 | 0.01 |
| **A549** | -0.0257 | 0.0462 | 0.00049 | N/A |
