# Supplementary material for: Nonenzymatic function of Aldolase A downregulates miR-145 to promote the Oct4/DUSP4/TRAF4 axis and the acquisition of lung cancer stemness
Source: Cell Death Dis. 2020 Mar 18;11(3):195. doi: 10.1038/s41419-020-2387-2 (PMC7080828; doi:10.1038/s41419-020-2387-2)
Supplement: Supplementary file 2 — Supplement Table 2 [file 41419_2020_2387_MOESM2_ESM.docx]

**Supplementary Table 2.** Predicted target site of microRNAs for POU5F1 obtained from miRcode.

| **microRNA family** | **Seed position** | **Seed type** | **Repeat** | **Primates** | **Mammals** | **Other vert.** |
| --- | --- | --- | --- | --- | --- | --- |
| miR-145 | chr1:155403346 | 7-mer-m8 | no | 11% | 0% | 0% |
| miR-148ab-3p/152 | chr1:155403971 | 7-mer-m8 | no | 22% | 0% | 0% |
| miR-153 | chr1:155403851 | 7-mer-m8 | no | 22% | 0% | 0% |
| miR-182 | chr1:155403424 | 7-mer-m8 | no | 22% | 0% | 23% |
| miR-101/101ab | chr1:155403200 | 7-mer-m8 | no | 11% | 0% | 0% |
| miR-31 | chr1:155403010 | 7-mer-m8 | no | 22% | 0% | 0% |
| miR-103a/107/107ab | chr1:155403585 | 7-mer-A1 | no | 22% | 0% | 0% |
| miR-375 | chr1:155403416 | 7-mer-m8 | no | 11% | 0% | 0% |
| miR-125a-5p/125b-5p/351/670/4319 | chr1:155403893 | 7-mer-m8 | no | 22% | 4% | 0% |
