# Supplementary material for: Nonenzymatic function of Aldolase A downregulates miR-145 to promote the Oct4/DUSP4/TRAF4 axis and the acquisition of lung cancer stemness
Source: Cell Death Dis. 2020 Mar 18;11(3):195. doi: 10.1038/s41419-020-2387-2 (PMC7080828; doi:10.1038/s41419-020-2387-2)
Supplement: Supplementary file 3 — Supplement Figure legends [file 41419_2020_2387_MOESM3_ESM.docx]

**Supplementary Figure Legends**

**Figure S1.** The correlation between the ALDOA mRNA levels and the IC50 values of the cisplatin treatment in various lung cancer cell lines from the CCLE microarray dataset.

**Figure S2.** Representative images of the density and morphology of the 2^nd^ population of spheroids for a napabucasin gradient concentration treatment with ALDOA expression in the CL1-0 cells.

**Figure S3.** Kaplan-Meier analysis of the expression of candidate microRNAs at concurrently low or high levels (or other levels) with the endpoint of overall survival probability of lung cancer patients obtained from the Kaplan-Meier Plotter database (n=195).

**Figure S4. (A)** Sequence and binding region between Oct4 and miR-145. **(B)** The qRT-PCR analysis of the expression of miR-145 in the ALDOA knockdown model with or without the miR-145 inhibitor treatment. In **B**, *GAPDH* was used as an internal control for loading. The significance of the differences in b was analyzed using Student’s *t*-test.

**Figure S5. A** qRT-PCR analysis of the mRNA expression of *APAF1* with or without the overexpression of an exogenous ALDOA-encoding gene in CL1-0 cells. **B** qRT-PCR results showing the mRNA expression of *PPARA* with or without the overexpression of an exogenous ALDOA-encoding gene in CL1-0 cells. In **A** and **B**, *GAPDH* was used as an internal loading control.

**Figure S6.** Heat-map showing the endogenous mRNA expression levels of ALDOA, DUSP4 and TRAF4 in lung cancer patients from the TCGA clinical cohort.
