# Supplementary figures and images for: Nonenzymatic function of Aldolase A downregulates miR-145 to promote the Oct4/DUSP4/TRAF4 axis and the acquisition of lung cancer stemness
Source: Cell Death Dis. 2020 Mar 18;11(3):195. doi: 10.1038/s41419-020-2387-2 (PMC7080828; doi:10.1038/s41419-020-2387-2)

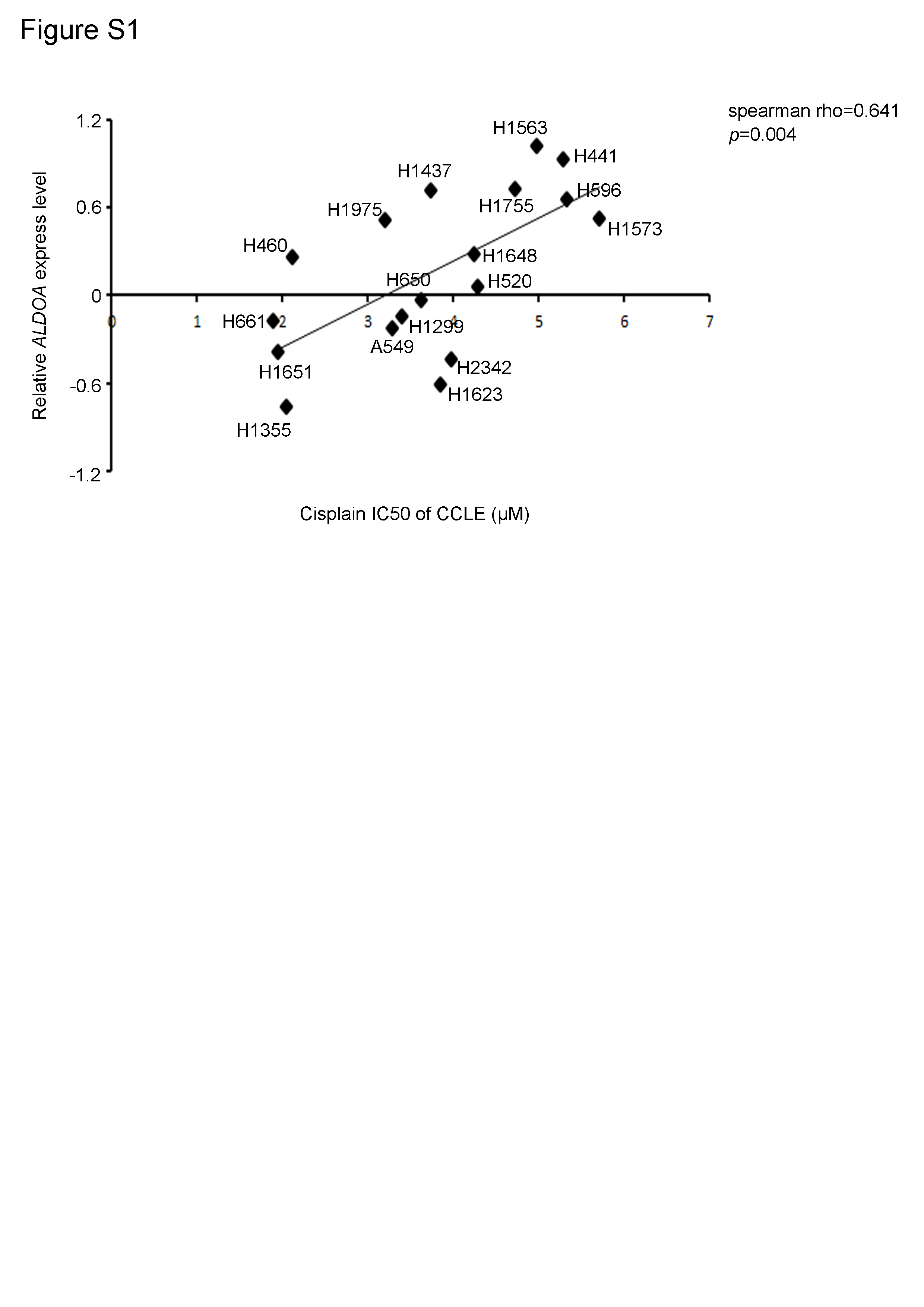

Supplement: Supplementary file 4 — Supplement Figure 1 [file 41419_2020_2387_MOESM4_ESM.tif]

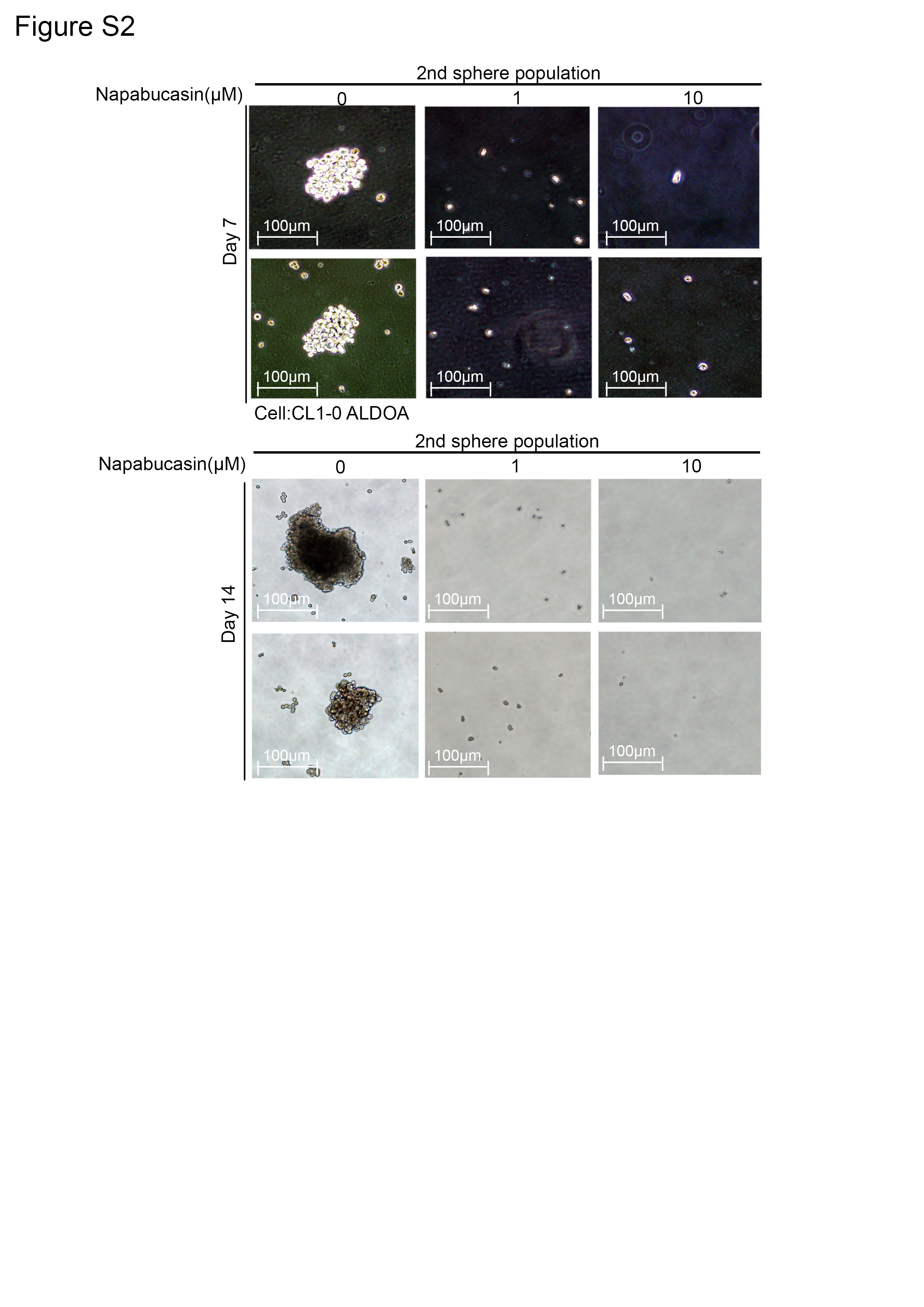

Supplement: Supplementary file 5 — Supplement Figure 2 [file 41419_2020_2387_MOESM5_ESM.tif]

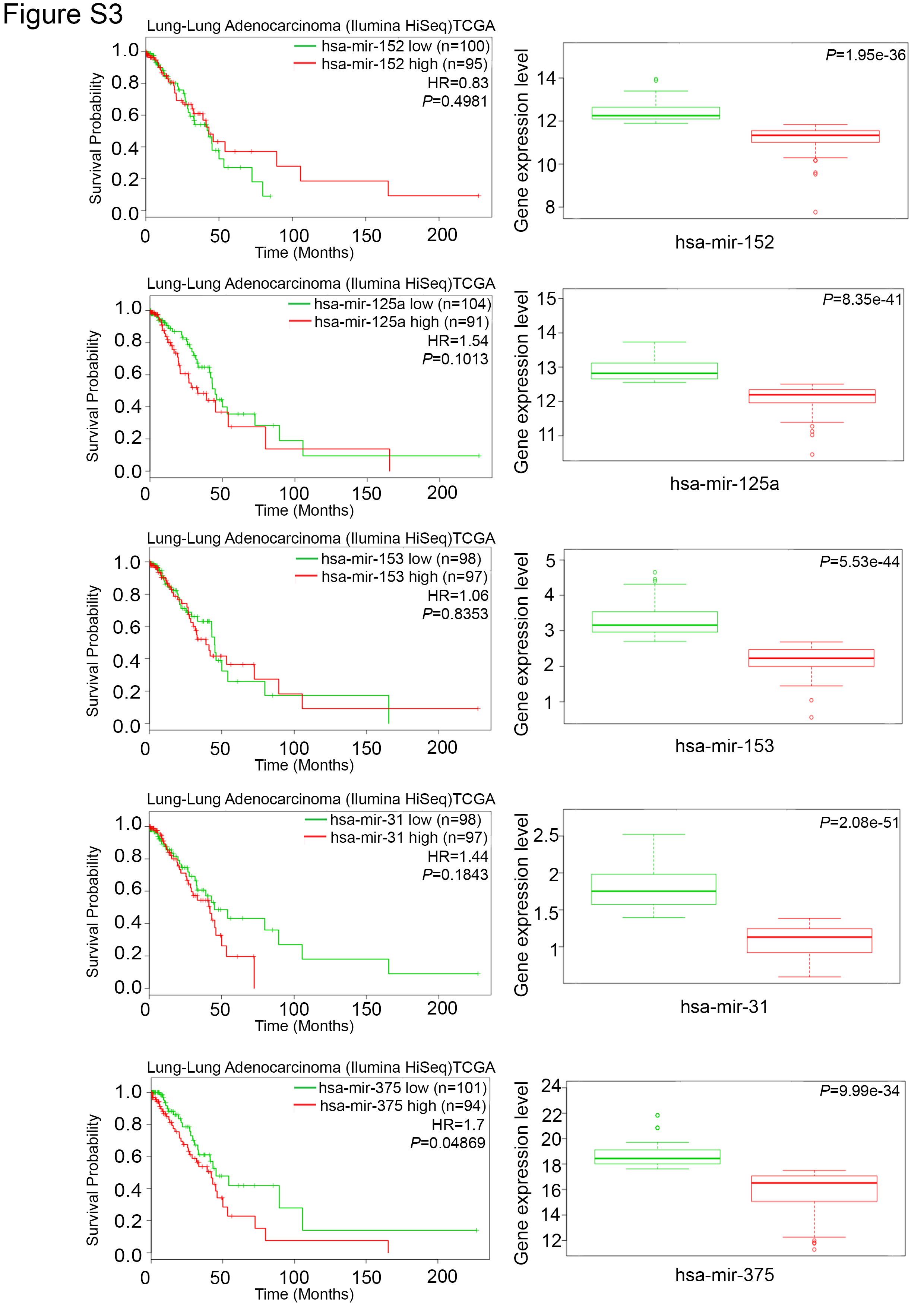

Supplement: Supplementary file 6 — Supplement Figure 3 [file 41419_2020_2387_MOESM6_ESM.tif]

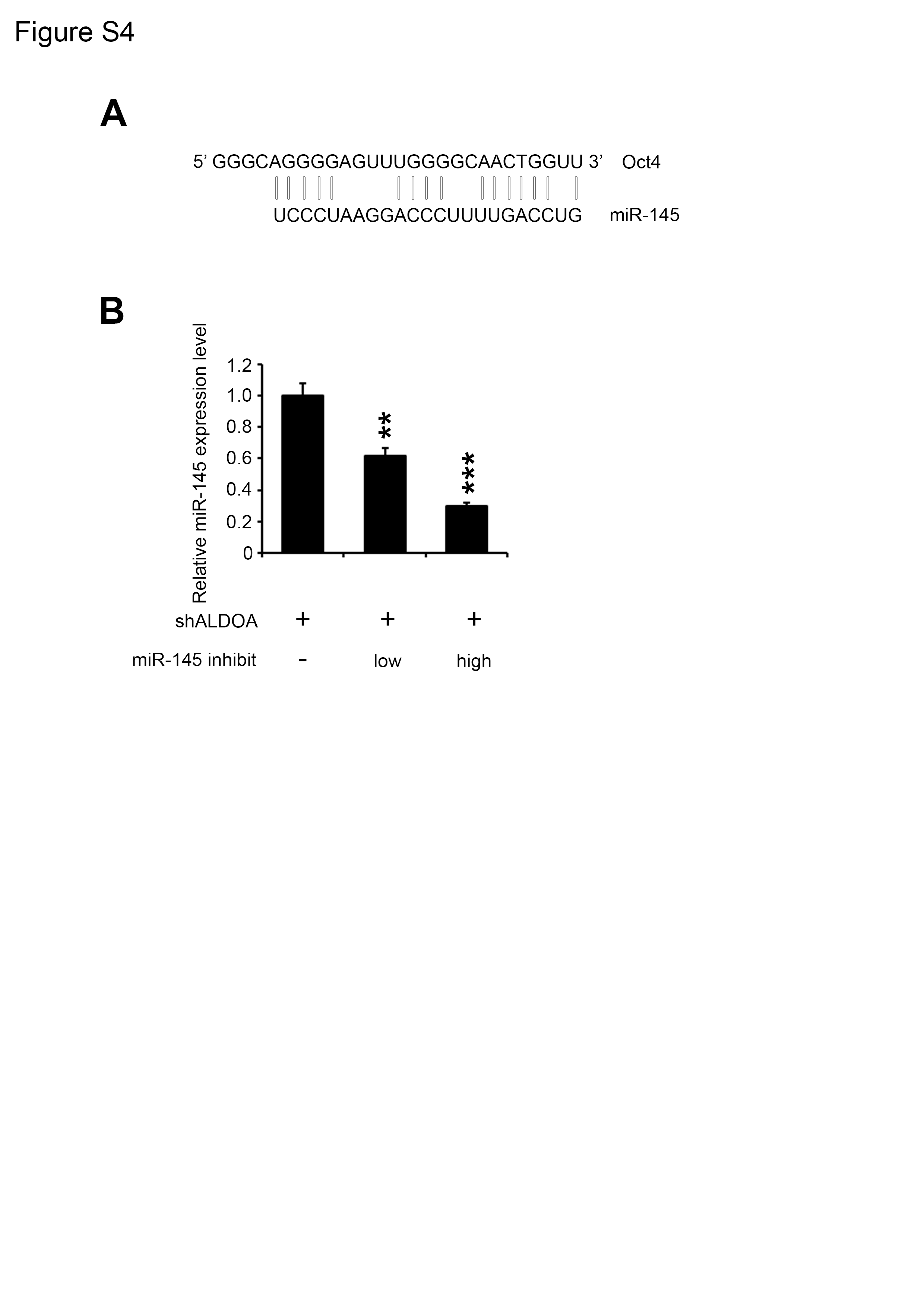

Supplement: Supplementary file 7 — Supplement Figure 4 [file 41419_2020_2387_MOESM7_ESM.tif]

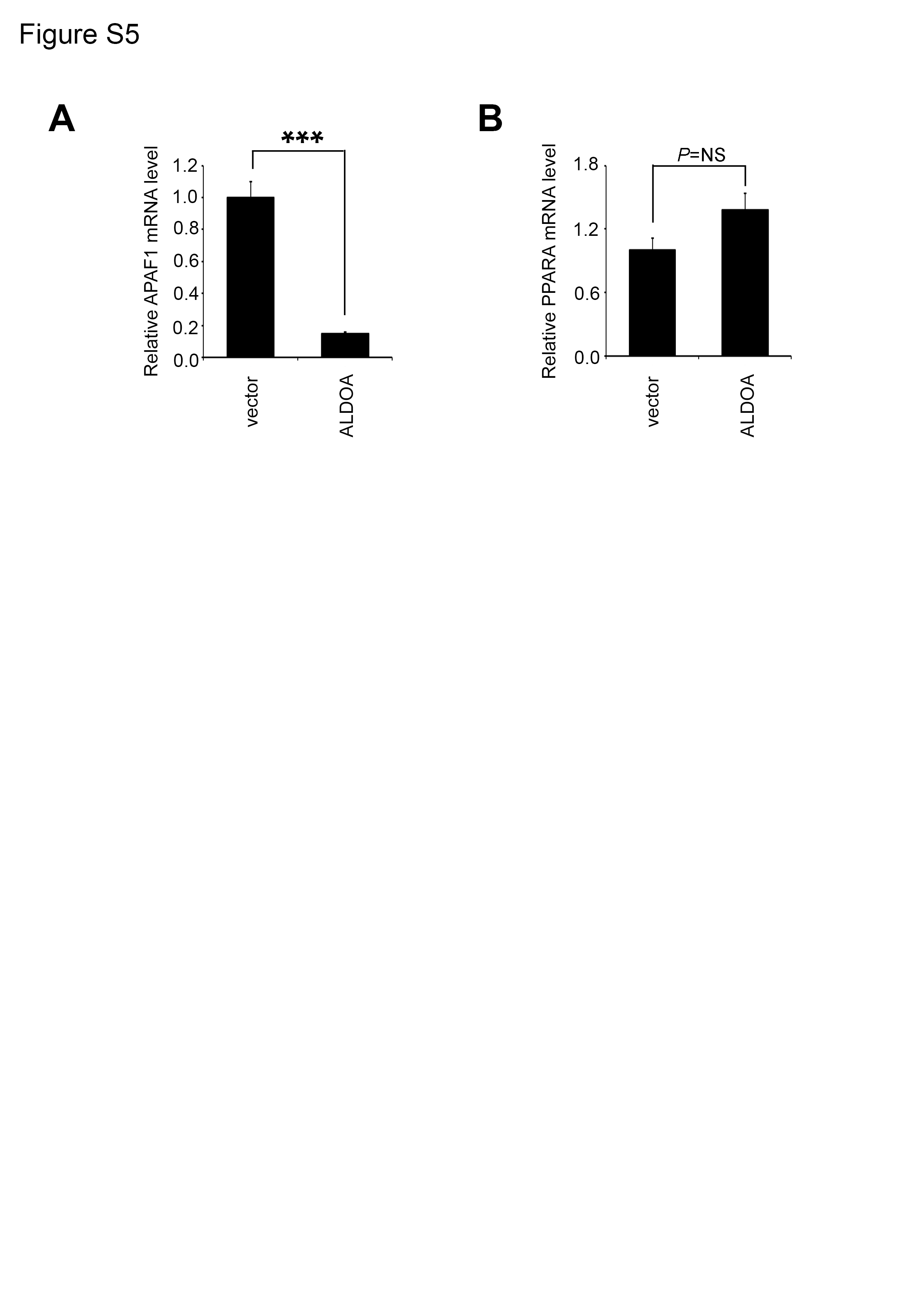

Supplement: Supplementary file 8 — Supplement Figure 5 [file 41419_2020_2387_MOESM8_ESM.tif]

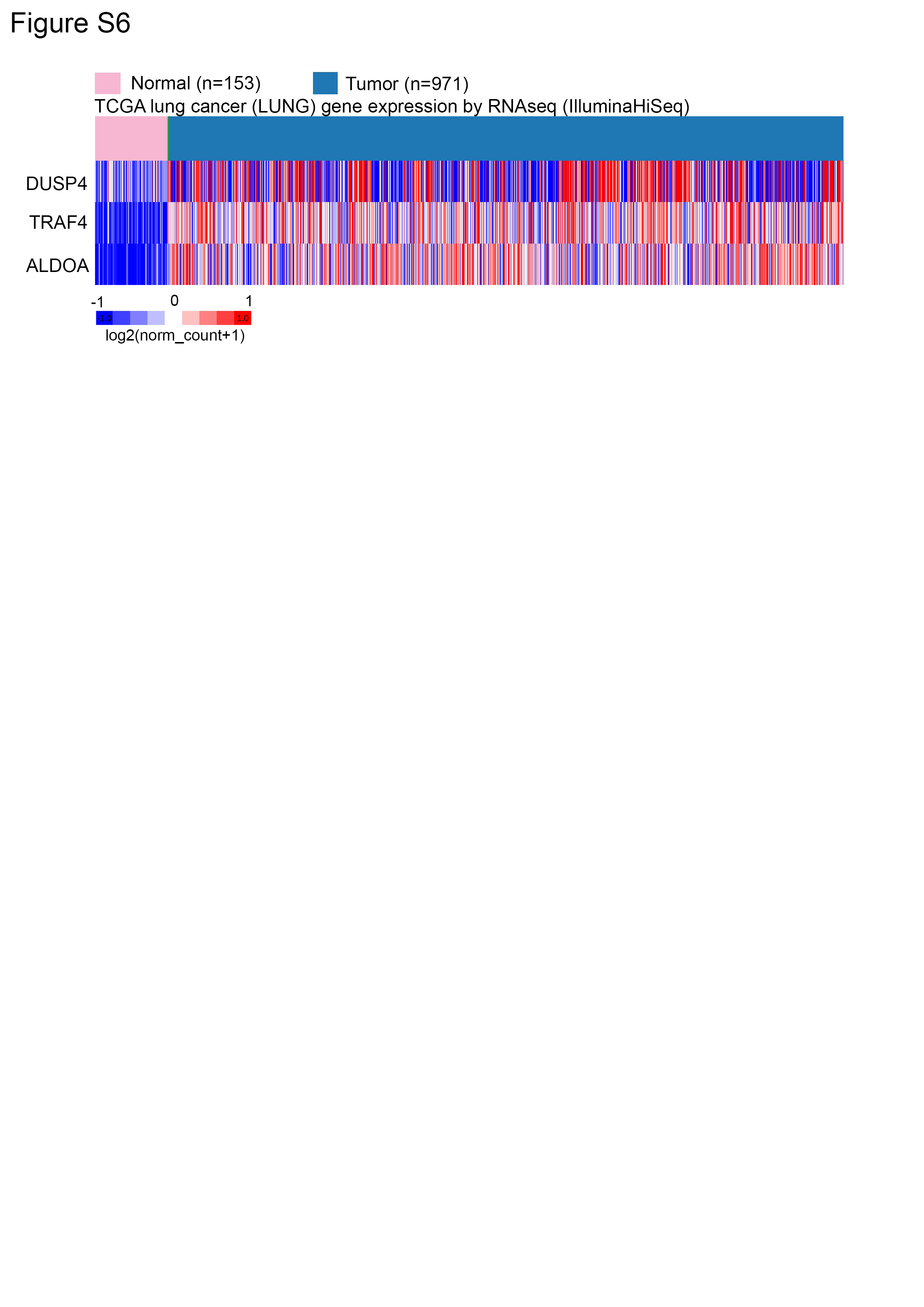

Supplement: Supplementary file 9 — Supplement Figure 6 [file 41419_2020_2387_MOESM9_ESM.tif]
